# The receptor RAGE: Bridging inflammation and cancer

**DOI:** 10.1186/1478-811X-7-12

**Published:** 2009-05-08

**Authors:** Astrid Riehl, Julia Németh, Peter Angel, Jochen Hess

**Affiliations:** 1 German Cancer Research Center, DKFZ-ZMBH Alliance, Division of Signal Transduction and Growth Control (A100), Im Neuenheimer Feld 280, 69120 Heidelberg, Germany

## Abstract

The receptor for advanced glycation end products (RAGE) is a single transmembrane receptor of the immunoglobulin superfamily that is mainly expressed on immune cells, neurons, activated endothelial and vascular smooth muscle cells, bone forming cells, and a variety of cancer cells. RAGE is a multifunctional receptor that binds a broad repertoire of ligands and mediates responses to cell damage and stress conditions. It activates programs responsible for acute and chronic inflammation, and is implicated in a number of pathological diseases, including diabetic complications, stroke, atheriosclerosis, arthritis, and neurodegenerative disorders. The availability of *Rage *knockout mice has not only advanced our knowledge on signalling pathways within these pathophysiological conditions, but also on the functional importance of the receptor in processes of cancer. Here, we will summarize molecular mechanisms through which RAGE signalling contributes to the establishment of a pro-tumourigenic microenvironment. Moreover, we will review recent findings that provide genetic evidence for an important role of RAGE in bridging inflammation and cancer.

## Introduction

**Figure 1:**
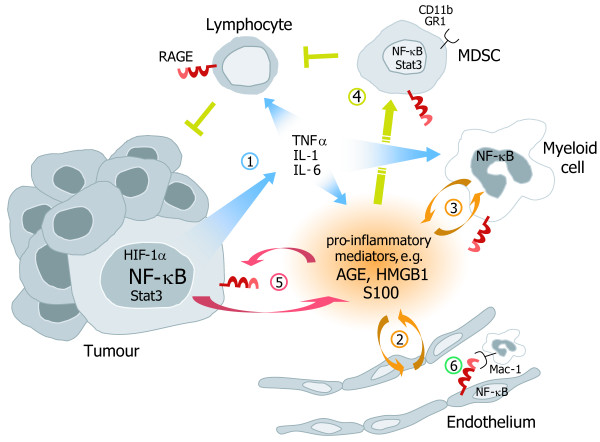
**RAGE function in inflammation-associated carcinogenesis**. RAGE is expressed in all cell types implicated in tumour formation, including tumour cells, endothelial cells, myeloid cells, MDSCs, and lymphocytes. Signalling pathways downstream of RAGE that are activated by the accumulation of its ligands (AGE, HMGB1, S100 proteins) regulate cellular interactions during neoplastic transformation and malignant progression: **(1) **A pro-tumourigenic microenvironment is established by the secretion of pro-inflammatory cytokines such as TNFα, IL-1, and IL-6, and the production of RAGE ligands. **(2, 3) **RAGE and RAGE ligands activate endothelial and myeloid cells resulting in the recruitment and accumulation of further myeloid cells, including MDSCs. **(4) **MDSCs inhibit T and natural killer cells leading to T cell tolerance and impaired anti-tumour immunity. **(5) **RAGE ligands and subsequent signalling also fuel tumour cell proliferation and survival by autocrine and paracrine feed-back loops. MDSC, myeloid derived suppressor cell; AGE, advanced glycation end products; HMGB1, high mobility group box-1; TNFα, tumour necrosis factor α, IL-1, interleukin-1; IL-6, interleukin-6.

Numerous findings, ranging from epidemiological studies to molecular analyses of mouse models, have highlighted a strong contribution of chronic inflammation to tumour development [1,2]. Irrespective of the cause of cancer-related inflammation, either driven by genetic alterations, tissue damage or arising from preceding infection, the generation of an inflammatory microenvironment supports tumourigenesis by promoting cancer cell survival, proliferation, migration, and invasion [3,4]. The generation of the pro-tumourigenic microenvironment strongly depends on the activation of several transcription factors, mainly nuclear factor-κB (NF-κB), signal transducer and activator of transcription 3 (Stat3) and hypoxia-inducible factor-1α (HIF-1α) [4]. These transcription factors regulate the expression of important cytokines, such as tumour necrosis factor α (TNFα), interleukin-1 and -6 (IL-1, IL-6) that are critically involved in the crosstalk between cancer cells and cells of the tumour stroma [5-7]. Importantly, these soluble factors and their respective receptors recruit and activate immune cells of lymphoid and myeloid origin and trigger signalling pathways resulting in the production of a large number of pro-inflammatory mediators in a positive feed forward loop [4,8] (Figure 1). Yet, the exact molecular mechanisms by which a pro-tumourigenic microenvironment is established and maintained, and subsequently promotes carcinogenesis remain largely elusive.

**Figure 2:**
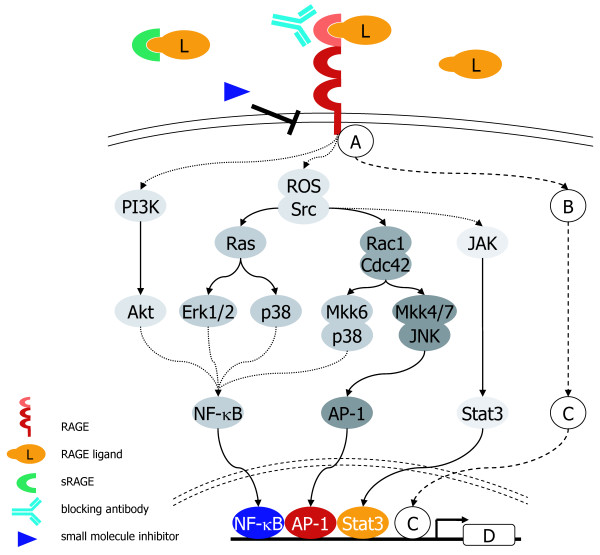
**Deciphering RAGE signalling for clinical interventions**. RAGE is known to interact with a broad spectrum of extracellular ligands and multiple signal transduction pathways have been shown to be directly (solid line) or indirectly (dotted line) activated upon RAGE ligation. Several tools for interference with RAGE-mediated signalling have been described: sRAGE, RAGE blocking antibodies and a small molecule inhibitor [21]. However, their successful use in clinical applications demands a comprehensive knowledge on intracellular signalling pathways and gene regulatory networks. So far, the unstructured C-terminal part of RAGE has hampered many approaches to find direct interaction partners within the cytosol (A). Other signalling molecules (B) besides PI3K, different MAPKs, Rho GTPases and ROS might be involved in functions of RAGE. On the level of transcriptional regulation, NF-κB, AP-1 and Stat3 have emerged as crucial targets of RAGE signalling, nevertheless other transcription factors (C) might be involved in regulation and function of RAGE as well. Finally, unravelling the RAGE-regulated genetic programme (D) will provide insight to the functional output of RAGE signalling. Cdc42, cell division cycle 42; Erk1/2, extracellular signal-regulated kinase 1/2; IκB, inhibitor of kappa B; IKK, inhibitor of kappa B kinase; JAK, Janus kinase; JNK, c-jun N-terminal kinase; MAPK, mitogen-activated kinase; MKK, mitogen-activated kinase kinase; NF-κB, nuclear factor kappa B; PI3K, phosphoinositide 3-kinase; Rac1, Ras-related C3 botulinum toxin substrate 1; ROS, reactive oxygen species; Stat3, signal transducer and activator of transcription.

RAGE (receptor for advanced glycation end products), a member of the immunoglobulin superfamily of cell surface molecules, interacts with diverse ligands, including not only advanced glycation end products (AGEs) and β-sheet fibrils, but also several members of the S100 protein family (S100B, S100P, S100A4, S100A6, S100A8/9, S100A11–13), high mobility group box-1 (HMGB1), and prions [9,10]. Despite data showing activation of multiple signalling pathways upon RAGE ligation (Figure 2), only little is known about proximal signalling events directly downstream of the receptor [11,12]. Furthermore, a broad range of evidence suggests that there is no single mode of RAGE activation by RAGE-binding proteins; first because of intrinsic properties of the different cell types, second the presence of three extracellular domains capable of ligand binding, and third the varied extent of ligand-induced RAGE oligomerization raising the idea of different adaptor proteins being recruited. The diverse properties of RAGE ligands, their interaction with the receptor, and tissue specific activation of signalling pathways are subject of several recent reviews [13-16].

Both, RAGE and its downstream signalling pathways are key players in cellular response to stress conditions, such as inflammation [14]. Induction of acute stress results in a transient synthesis and release of RAGE ligands, which induce an innate repair mechanism leading to a rapid but resolving response. However, in an environment of chronic stress with accumulation of ligands, RAGE triggers the conversion of acute cellular activation into sustained cellular response and tissue dysfunction [14,17].

### Regulation and function of RAGE in inflammation

Increased expression of RAGE has been documented in a variety of acute and chronic inflammatory diseases, such as septicaemia, rheumatoid arthritis, osteoarthritis, arteriosclerosis, chronic renal disease, inflammatory bowel disease, vasculitis and late diabetic complications (see references in [18]). In the past, numerous findings have advanced our knowledge on the molecular mechanisms through which RAGE could contribute to cellular processes of adaptive and innate immune responses [14,15]. First, RAGE expression has been found on most key cell types linked integrally to an immune response, i.e. monocytes/macrophages, neutrophils, dendritic cells, as well as T and B lymphocytes [19-21] (Figure 1). Second, RAGE is expressed on endothelial cells and acts as an adhesion receptor for leukocytes by physical interaction with β2 integrin Mac-1. Interestingly, RAGE-Mac-1 interaction is induced by pro-inflammatory RAGE ligands *in vitro *and promotes leukocyte recruitment in mouse models of inflammation [22,23]. Third, among the broad spectrum of extracellular ligands that trigger RAGE signalling, many have been functionally linked with acute and chronic immune responses [24-26]. Fourth, engagement of RAGE induces activation of the transcription factor NF-κB and some of its downstream target genes that are well-known regulators of the adaptive and innate immune system [10]. It is worthwhile to note that RAGE also exhibits a functional NF-κB binding site in its proximal promoter and has been shown to be a direct target gene of NF-κB signalling [27]. This might explain, at least in part, why RAGE expression is absent or very low in normal adult tissues, except for the lung, and increases under stress and pathological conditions [28-30]. Furthermore, ligands of RAGE have been shown to accumulate and trigger intracellular activation of NF-κB at sites of tissue damage and inflammation [31]. This interconnected signalling enables a cycle of prolonged cellular response supporting the establishment of chronic tissue alterations [10]. In addition, sustained NF-κB signalling in response to RAGE activation is also mediated by *de novo *RelA (p65) mRNA synthesis, which results in a growing pool of transcriptionally active NF-κB, overriding endogeneous negative feedback mechanisms [32].

Important insights regarding the specific function of RAGE in pathological conditions affected by innate and adaptive immune responses have been obtained from mouse models in which Rage signalling was inhibited. This was achieved by (i) administration of blocking antibodies or recombinant soluble RAGE (sRAGE), a truncated form that acts as a potent decoy-receptor for ligands, (ii) expression of a dominant-negative *Rage *(*DN-Rage*) transgene in genetically modified mice, and (iii) complete or tissue-specific deletion of *Rage *(*Rage*^-/-^). Treatment with sRAGE and expression of *DN-Rage *interfered with inflammatory conditions and revealed therapeutic effects in mouse models of diabetic atherosclerosis, delayed-type hypersensitivity (DTH), neuroinflammatory disorder, colitis, periodontitis, and others [18,33]. However, it is important to note that sRAGE affects immune responses even in the absence of functional Rage, leading to the assumption that its beneficial effects in mouse disease models are not solely caused by preventing ligand engagement of Rage but also of other receptors [34]. Studies with *Rage*-deficient mice provided further *in vivo *evidence for the central role of RAGE in initiation and perpetuation of immune responses [18]. Although *Rage*^-/- ^mice display a mild pro-inflammatory phenotype characterized by a moderately increased basal NF-κB activation and cytokine expression, they are protected from lethal septic shock in a model of cecal ligation and puncture (CLP), and survive in a *Listeria monocytogenes *infection model [34,35].

While an important function of RAGE signalling is widely accepted in myeloid cells affecting primarily the innate immune response, opposing data were published in the context of the adaptive immune response. On the one hand, Liliensiek and colleagues reported that *Rage*^-/- ^mice showed unchanged onset and progression of disease in models of DTH and experimental autoimmune encephalomyelitis (EAE), suggesting a normal T cell-dependent immune response [34]. On the other hand, specific loss of Rage function in T lymphocytes impaired priming of adaptive immune responses *in vivo *[21,36].

### Regulation and function of RAGE in cancer

RAGE expression has been detected in a variety of human tumours, including brain, breast, colon, colorectal, lung, prostate, oral squamous cell, and ovarian cancer, as well as lymphoma and melanoma [37]. Although our knowledge on the molecular function of RAGE during neoplastic transformation and malignant progression is limited, recent experimental data ranging from *in vitro *analyses to studies in mouse models and clinical data support a direct link between RAGE activation and proliferation, survival, migration, and invasion of tumour cells [37,38]. Moreover, gene expression studies with samples of human patients and mouse tumour models revealed overexpression of RAGE ligands in most types of solid tumours [24,39]. Taguchi and colleagues found that blockade of the Hmgb1-Rage axis suppressed tumour growth in two independent mouse models, and thereby provided for the first time experimental data for an *in vivo *function of Rage during cancer development [40]. Yet, in contrast to the concept of RAGE as a potent inducer of tumour growth and malignant conversion, few reports raised the perception that it also has tumour-suppressive functions in distinct cell types. As an example, RAGE is highly expressed in lung tissue, especially at the site of alveolar epithelium, and its expression is significantly reduced in lung carcinomas [41,42]. This suggests that lung cancer progression may be enhanced by loss of RAGE function and indeed re-expression of RAGE in lung tumour cell lines reduced their proliferation and revealed diminished tumour growth in athymic mice [43-45]. In addition, functional inactivation of RAGE in myoblasts resulted in reduced myogenesis and enhanced proliferation and invasion *in vitro *as well as increased tumour growth *in vivo *[46,47]. Nevertheless, tissue-specific differences in levels of RAGE expression, its splice variants, and ligands give room for the speculation that RAGE might exhibit tumour-suppressive functions in tissues characterized by constitutive RAGE expression, while it fulfils a tumour-promoting role in tissues with inducible RAGE expression.

### RAGE in inflammation-associated cancer

Given the evidence from a large body of experimental data that signals downstream of RAGE can fuel chronic inflammatory conditions (see above), one could speculate on its potential impact on creating a microenvironment that is ideal for neoplastic transformation and malignant conversion (Figure 1). Indeed, the usage of *Rage*-deficient mice (*Rage*^-/-^) in well-established mouse models of inflammation-associated carcinogenesis, such as chemically induced skin carcinogenesis and colitis-associated cancer, provided direct genetic evidence for a novel role of Rage signalling in linking chronic inflammation and cancer [20,48]. Chemically induced skin carcinogenesis is one of the best-established *in vivo *models to study the multistage nature of tumour development. Moreover, it represents a classical inflammation-associated tumour model, as its promotion phase solely depends on repeated topical treatments with the phorbol ester 12-O-tetradecanoylphorbol-13-acetate (TPA), a potent inducer of dermal inflammation [49]. We could show that *Rage*^-/- ^mice were almost resistant to 9,10-dimethyl-1,2-benzanthracene (DMBA)/TPA-induced skin carcinogenesis and that the few tumours that developed in the absence of Rage were smaller in size, less progressed, highly differentiated, and hyperkeratotic [20]. Importantly, dramatically decreased levels of pro-inflammatory mediators (e.g. *Ptgs2*, *S100a8*, *S100a9*, and *macrophage inflammatory proteins*) and reduced numbers of infiltrating immune cells accompanied impaired tumour formation in *Rage*^-/- ^animals. These data suggest a severe defect in sustaining inflammatory conditions during the promotion phase. One intriguing finding of the study was the ability of Rage to induce its own ligands, as we found strongly induced S100a8 and S100a9 expression in epithelial cells only in the presence of Rage. Hence, the S100-Rage axis might generate a feed-forward loop that further aggravates an inflammatory environment [20]. In addition, S100A8/S100A9 could directly induce proliferation of tumour cells via RAGE ligation, as was shown recently in a cell culture model [50]. Bearing in mind the importance of the pro-inflammatory microenvironment in tumour development, it is interesting to note, that bone marrow chimera experiments revealed Rage expression on immune cells, but not keratinocytes or endothelial cells, to be essential for innate immune cell recruitment and induction of epidermal hyperplasia *in vivo *[20]. This is in line with published data on a model of HMGB1-induced peritonitis [23]. Nevertheless, Rage expression on keratinocytes might also contribute to the transformation and malignant progression of epithelial cells, and it will be a challenge for the future to investigate cell type-specific *Rage *knockout mice using the DMBA/TPA tumour model.

In line with the data obtained from the model of inflammation-associated skin carcinogenesis, Turovskaya and colleagues demonstrated a crucial role for Rage in a mouse model of colitis-induced cancer (CAC) [48]. Although Rage was dispensable for initial acute inflammation, *Rage*^-/- ^mice showed defects in the transition to chronic inflammatory conditions and revealed significantly less tumours. Interestingly, impaired CAC was not only observed in *Rage*^-/- ^mice, but also in *wt *mice after administration of mAbGB3.1, a monoclonal anti-carboxylate glycan antibody [48]. Glycosylation of the extracellular part of RAGE significantly increases receptor affinity for HMGB1 and S100 proteins. In addition, carboxylated glycans were found on a subset of RAGE on colon cancer cells [51,52].

A common feature of both mouse models, chemically induced skin carcinogenesis and CAC, was a reduced number of Gr1^+^CD11b^+ ^myeloid precursor cells in the absence of Rage function, implying that RAGE signalling also contributes to the development and recruitment of myeloid-derived suppressor cells (MDSCs). Accumulation of MDSCs is one of the major immunological abnormalities in cancer resulting in T cell tolerance and suppression of anti-tumour immune response [53] (Figure 1). The molecular mechanisms of this phenomenon remain largely undefined, however, a recent study demonstrated that tumour-induced and Stat3-dependent up-regulation of S100a8 and S100a9 proteins in myeloid precursor cells are necessary for the accumulation of Gr1^+^CD11b^+^MDSCs [54]. Moreover, Sinha and colleagues found that S100a8/S100a9 produced and secreted by tumour cells binds to Rage on MDSCs and promotes their migration and accumulation through NF-κB signalling pathways [55].

## Conclusion and perspective

In summary, the published data propose that RAGE signalling drives the strength and maintenance of an inflammatory response and serves as a key player in bridging chronic inflammation and cancer. Accordingly, compelling data from diverse animal models have provided a rationale for targeting RAGE as a novel strategy for clinical interventions, and thereby, hit two important compartments during carcinogenesis: the transformed tumour cells as well as the pro-tumourigenic microenvironment. Several approaches have been considered to pharmacologically antagonize RAGE or RAGE ligand-induced pathologies, including cancer [18,35]. Despite these promising results, the tumour-suppressive functions of RAGE that were observed in specific tissues raise concerns against the therapeutic use of RAGE inhibitors and hamper a systemic application of such. Furthermore, no long-term studies using sRAGE, blocking antibodies, or small molecule inhibitors are available yet, necessary to appraise potential side effects. This is of particular interest, as RAGE has been shown to be important for cellular repair and tissue regeneration/homeostasis [14].

Altogether, there is an urgent need for a better and comprehensive understanding of the molecular mechanisms through which RAGE executes its beneficial function during acute responses and tissue homeostasis or contributes to a feed-forward signalling in severe chronic disorders, including cancer. Despite the fact that RAGE has been identified almost two decades ago, it still retains a lot of its mystery (Figure 2). Therefore, systemic and genome-wide approaches will be required to solve the complexity of RAGE-dependent signalling pathways and gene regulatory networks, also with respect to the functional crosstalk with other receptors such as Toll-like receptors [56]. Eventually, the availability of a large variety of informative mouse models and some distinct downstream target genes will be a promising starting point for novel drug development and drug assessment in the near future.

## Competing interests

The authors declare that they have no competing interests.

## Authors' contributions

AR selected the relevant literature, was involved in interpretation and discussion of published data, drew the figures and wrote the manuscript. JN drafted parts of the manuscript and made substantial contribution to the interpretation of published findings. PA was involved in revising the manuscript for important intellectual content. JH drafted parts of the manuscript and was strongly involved in data interpretation and critical discussion of the manuscript. All authors read and approved the final manuscript.
